# “It Is Our Exercise Family”: Experiences of Ethnic Older Adults in a Group-Based Exercise Program

**Published:** 2007-12-15

**Authors:** Basia Belza, Kuan-Chun Chiang, Leslie Seman, Jenny Hsin-Chun Tsai

**Affiliations:** University of Washington; School of Nursing, University of Washington, Seattle, Washington; School of Nursing, University of Washington, Seattle, Washington; School of Nursing, University of Washington, Seattle, Washington

## Abstract

**Introduction:**

EnhanceFitness (EF) (formerly the Lifetime Fitness Program) is an evidence-based community exercise program for older adults. From 1998 to 2005, participation of ethnic older adults increased significantly. However, little research is available about what ethnic older adults want or need to continue participation in exercise programs. The purpose of this study was to examine how physical environment, social environment, and individual biology and behavior influence adherence to exercise for ethnic older adults participating in EF.

**Methods:**

Six focus groups were conducted with 52 older adults participating in EF. Facilitators asked questions about factors that helped participants continue exercising in EF. Interviews were audiotaped and transcribed. Transcripts were systematically reviewed using content analysis.

**Results:**

Focus group participants were Chinese (n = 21, 40%), African American (n = 18, 35%), white (n = 10, 19%), and Japanese (n = 3, 6%). Mean (SD) age was 76 years (7.4). Participants had, on average, participated in EF for 44 months (SD = 37.8). Results revealed four themes related to adherence. First, environmental factors that promoted adherence were location of the classes, transportation, weather, and the facility. Second, design of the exercise program that encouraged adherence included exercise content and type of delivery. Third, social support factors that encouraged adherence were the socializing and support between class participants and support from family, health care providers, and the class instructors. Finally, individual factors that encouraged adherence were personality traits and feelings, past physical activity experience, health benefits, and mental stimulation.

**Conclusion:**

Findings from this study suggest strategies for developing community-based physical activity programs for older adults from ethnically diverse communities.

## Introduction

Regular participation in exercise generates physical and psychological benefits and is an essential component for healthy aging (1-5). A structured group exercise program offers additional psychosocial benefits for older adults (6,7). However, few studies target older adults from different ethnic groups. Research on how to attract ethnically diverse older adults into exercise programs is also lacking (8,9). More research is needed about exercise in older ethnic adults (9) who are also at greater risk of morbidity (10). Studying the types of exercise programs that older adults prefer and how these match personal needs, values, and circumstances will help researchers, health care professionals, and providers of aging services to design and promote successful programs. In our study, we used an ecologic model to examine the perspectives of ethnic older adults and to explore factors that promote exercise adherence. Participants offered a rich variety of information, outlooks, and outcomes extending beyond the literature. 

### Ecologic model

Researchers have addressed individual and social factors that influence long-term exercise participation of older adults (6,11,12). However, little research examines the influence of environmental factors such as the physical environment and availability of well-designed exercise programs. The determinants of the health "ecologic model," as discussed in *Healthy People 2010 *(13), illustrate the individuals' health transactions with their physical and social environments and can be used to study exercise behavior . Satariano and McAuley (14) describe the ecologic model by noting that "health depends on the dynamic interaction of biological, behavioral, social, and environmental factors that interact over the life course of individuals, families, neighborhoods, and communities" (pp. 184-5). A feature of this model is that physical environment, social environment, and individual variables of behavior and biology all influence health in an interactive manner (15). The ecologic model was used as a framework in this study to examine older ethnic adults' adherence to exercise. Although this model includes six determinants that influence an individual's health behavior, we discuss three that were integral to our study: 1) physical environment, 2) social environment, and 3) the individual's biology and behavior.

Physical environment describes the settings in which people live, exercise, and interact. Establishing physical activity programs in senior centers, community centers, churches, and retirement homes was key to increased participation (9,16). Participation in an exercise program depends not only on the facility's convenience but also on its safety, attractiveness, and cost of participation (9,10). Overall, the association of physical environmental factors with older ethnic adults' exercise patterns remains a neglected area of study (9). A study involving seven ethnic focus groups identified environmental barriers to exercise, including weather, neighborhood safety, fear of crime, program costs, and inadequate availability and reliability of affordable transportation (17).

Social environment refers to interactions with family, friends, and others in the community as well as cultural customs. Social support from family, friends, program staff, members of the exercise group, or health care providers increases exercise participation (8). The support of community, family members, and friends is especially important to ethnic older adults (10,17). The social networks within exercise groups enhance individual self-efficacy and adherence to, and persistence in, the exercise program (18). When the participants like their instructors, they are more motivated to come to class (19).

Individual behavior and biology refers to a person's responses, traits, characteristics, feelings, past experience, and health. Self-efficacy — an individual's belief in her or his ability to successfully perform a specific behavior (20) — is a well-known trait that determines exercise behavior in different populations (14,18). Individuals with strong self-efficacy are more likely to persist with a behavior. This trait influences exercise behavior by moderating behavioral change such as starting an exercise class, determining whether a particular exercise will be attempted, determining the degree of persistence if the exercise is difficult, and determining the success or failure of completing the class (21). Few studies examine self-efficacy and exercise in ethnic older adults. In a study of African American women with arthritis, self-efficacy was the most consistent factor affecting physical activity behavior (22). Motivation and willpower were identified as very important by three Latino focus groups in starting and adhering to an exercise program (23). Attitudinal and psychological beliefs such as a wish to improve health, fitness, and appearance through exercise are held by older as well as younger women (9). Feeling good and enjoying physical activity contribute to higher self-efficacy in older adults (18). Enjoyment and improvement of mental and physical health were valued by older African American women (24).

### The EnhanceFitness (EF) Program

EnhanceFitness (EF) (formerly Lifetime Fitness Program) is an evidence-based community exercise program for older adults (16). The EF program is offered in hourly sessions 3 times per week and includes strength, endurance, balance, and flexibility exercises (25). The program is now offered in 53 locations at senior centers and community centers in Seattle and in King County, Washington, and has 177 sites in 18 states. In recent years EF classes have been established for African American, Hispanic, Hmong, Korean, Filipino, Somali, Vietnamese, American Indian, and Chinese older adults in Seattle. According to EF program director S. J. Snyder, the percentage of EF participants from ethnic communities increased from 1% in 1998 to 25% in 2005 (personal communication, May 2005). Despite the significant increase in ethnic participation, little is known about the factors that may have drawn ethnic older adults to EF.

To address the literature gap, we examined how physical environment, social environment, and individual biology and behavior influenced adherence to exercise among ethnic older adults participating in EF. Our goal is to use the information to generate effective strategies to promote  adherence to exercise programs among ethnic older adults. The information will also be useful for future EF program evaluation and development.

## Methods

### Design and sampling

Six focus groups were conducted in October and November 2005 in two urban neighborhood senior centers and a Chinese church in Seattle. With the assistance of the EF instructors and program coordinator, participants were recruited from three EF exercise sites that had predominantly ethnic participants. Inclusion criteria were participation in the EF program for at least 1 month, being aged 55 or older, and the ability to read and speak English or Cantonese. The ability to read and speak Cantonese was included because one site was a Chinese church in which half the people spoke Cantonese and the other half spoke English. Approval to conduct the study was obtained from the University of Washington Institutional Review Board. Investigators complied with the approved protocol in all stages of the study.

### Data collection

Five focus groups were conducted in English and one in Cantonese. The Chinese investigator in this study led one English focus group and the white investigator led the other four English focus groups. Both investigators were graduate nursing students who were trained in focus group facilitation and who had worked with older adults. A Cantonese-speaking Chinese interpreter skilled in focus group interviews conducted the Cantonese-speaking focus group. An interview questionnaire developed by the investigators was used to explore the influence of physical environment, social environment, and individual behavior and biology on exercise adherence among older ethnic adults (Table 1). Each focus group had 8 to 10 participants and lasted, on average, 70 minutes. All groups were audiotaped. Participants were offered lunch or a gift certificate for participation. Demographic data were collected using a 7-item demographic questionnaire.

### Data analysis

Audiotapes were transcribed into Microsoft Word documents. The audiotape of the Cantonese-speaking focus group was translated into English and transcribed by the interpreter who led the group. Content analysis, a process of organizing and integrating narrative, qualitative information according to emerging themes and concepts (26), was used to develop themes. Guided by the ecologic model (13), the investigators independently read and coded each transcript. Contrasts and similarities of themes within and across groups were examined; a final set of themes were merged and categorized to capture aspects of the ecologic model and those not included in the model. The first and second authors discussed relevance of the themes, quotes, and definitions with the third and fourth authors, who have expertise in qualitative research. Demographic data were described with descriptive statistics.

## Results

### Sample

Fifty-two adults, mean age 76 years (SD = 7.4; range, 62–96 years), participated in the study. They were Chinese (n = 21, 40%), African American (n = 18, 35%), white (n = 10, 19%), and Japanese (n = 3, 6%). Eighty-five percent were female. All participants lived in an urban environment. They had, on average, participated in EF for 44 months (SD = 37.8; range, 2–96 months) and attended EF classes 2 to 3 times per week. Ninety percent indicated that they were highly confident they would continue to attend this EF program for the next 6 months (Tables 2 and 3).

### Themes

The identified themes included physical environment, the design of the EF program, social environment, and individual behavior and biology (Table 4).

#### Physical environment

*Location.* Participants at the two senior centers and the church were satisfied with the neighborhood location of EF classes. Some participants had attended other exercise classes but preferred the neighborhood location closer to their homes because they could participate in activities at the church and senior centers other than exercise.

*Transportation, weather, and facility.* Most participants drove to the exercise classes; some walked, carpooled, or rode the bus. Many commented that bus service was nearby. Weather did not appear to be a drawback, except on the rare occasion when it snowed and the class site closed. Participants were satisfied with the facilities where the classes were held. They appreciated the church location because it is large and has a nice indoor gym.

#### Design of the EF Program

Exercise content, program delivery (e.g., timing, cost), and physical performance evaluation emerged as themes. The participants enthusiastically supported the variety of exercise and the complete body workout that they were getting in class. The exercise content was perceived as systematic, senior-specific, and easy to follow. One participant described the exercises as "covering all the joints from the upper body to lower body through range of motion, balancing, weight-lifting, and aerobics." They valued this exercise program because it met their health needs. One participant said, "The weights help us to get the benefit from exercising." The physical performance evaluations that were conducted every 4 months provided feedback to participants about their progress. The morning timing of classes was preferred, as it provided a reason to get up and out of bed. The low- or no-cost EF classes were appreciated and attracted participants to join.

#### Social environment

*Socializing and support among exercise class participants*. Most of the participants enjoyed socializing, building friendships, and being with peers. For some, it was their main social outlet: "It is a way for me to stay in touch with the world, with my community." Joining the exercise program was especially important to those who lived alone: "I live alone and as long as I belong to the exercise class, that is something to make me get up and get dressed and get out." Several participants did activities such as line dancing, shopping, and eating lunch with friends from class.

Having a network of peers was another reason that participants enjoyed the class. Participants helped each other by sharing rides, phoning each other, and demonstrating caring, and enjoyed sharing common issues with their peers. One participant mentioned, "I get to talk, too. If I have a problem, I discuss it and see what they would do about it."

The exercise class itself formed a social network that provided participants material, verbal, emotional, and sometimes spiritual support. One participant commented, "It is our exercise family!"

*Support and influence from family*. Family influence and support was another theme. Participants in all groups talked about support from their children, spouses, and other family members for their participation in EF. For instance, their family kept track of them to see if they attended the exercise class. Some families tried not to make plans on their exercise days; others helped them to find out about this program and encouraged them to join. One participant was happy when his wife joined the EF class after he did. A 96-year-old participant said, "My children, every time I get tired and want to stop and lay off, 'no you go on.' They drive me here."

The desire to stay healthy for their family was a strong motivation. Participants exercised because they did not want to be a burden on their family. Others wanted to stay healthy to help take care of a family member or to see their grandchildren. One Chinese participant said, "On my birthday, my grandson gave me 100 pennies and said I want you to live 100 years. I decided to live longer to see my grandchildren. That is why I have a strong desire. I do that for my family."

*Support from health care providers*. Many participants started the exercise program because of their health care providers' encouragement or referrals. One participant said, "When I retired in 1995, my doctor recommended EF and here I am. I have been here for 10 years." Participants said that their doctors' positive feedback and supportive attitude helped them to keep exercising. One participant shared: "My doctor that I see for some years, his opening question usually is, are you still exercising?"

*Support and influence from exercise instructors.* Participants perceived their instructors as people who are enthusiastic, motivating, and who make exercise fun and like older adults. Participants in every focus group said that their instructors were a major reason they continued to participate in EF. They appreciated their instructors' knowledge and expertise and liked the personal help that the instructors provided: "He goes from person to person to show them, to help them, you know, because so many of us are stiff and we are not doing it quite right." When people were absent from class, instructors phoned to check on them. One participant said: "She motivates us, she makes it fun. I hope she never resigns."

*Shared language and religion.* The Chinese participants all attended the same church, which was where their EF class was held. Several participants from this class stated that they attended EF because their class was conducted in Cantonese and they could understand the instructor's directions. Some English-speaking Chinese participants commented that they enjoyed being with their Cantonese peers and learning the language. Participants mentioned that they wanted to support their church by attending EF classes there.

#### Individual behavior and biology

*Personality traits and characteristics.* Participants mentioned several personality traits and characteristics that helped them keep exercising. One participant said, "I am very competitive"; another said, "What I enjoy is the competition with myself. I like the healthy feel of competition." Participants identified perseverance, a positive attitude, commitment, and confidence as traits that kept them coming to class. A male participant, for instance, said that his "sense of humor" got him through class and his life. A female participant explained, "Your self-confidence is reinforced when you come here. The sense of well-being and ability and you can do things. Sometimes I forget I'm 86 years old."

Independence and liking to have structure in daily life were commonly mentioned personality traits. Many participants wished to maintain their independence as they aged, and they regarded the EF classes as a way to do so. An African American participant's comment illustrated this idea well: "You want to be independent and you want to be self-sustaining, so this is a drive and I think it is ultimately what everybody is thinking about, because I want to be on my own. I want to be independent. You want to take care of yourself as long as you can. That is the whole game."

Liking to have structure in daily life was shared by many participants. "I enjoy the routine of 'must get up, must get out'," said a participant. Most agreed that the structured format of the class was an incentive to them to get out of their houses and into a situation where they exercised with each other.

*Personal feelings.* Personal feelings that motivated exercise varied. Boredom with her new retired life was the reason that one female participant joined the exercise class. A guilty conscience kept a few participants coming to class. As one man said, "I feel guilty if I don't come. I have to come up with some good excuses." Pride was another personal feeling mentioned by a participant. She said, "I think it was a matter of pride for me. I want to keep active, I want to keep healthy, and keep in shape. I keep denying, you know, some of the forces of my excess weight, but it still is a matter of pride to at least try and do something."

For many participants, the cheerful group dynamic was a reason to come to the exercise class.

*Past physical activity experience*. Many participants mentioned other physical activities that they currently did or had done in the past as factors related to their participation in EF. The activities included walking, doing exercises at home, baby-sitting, aerobic dancing class, line dancing, yard work, housework, bicycling, walking dogs, Tai Chi, and acting as a caregiver. Among them, walking was most frequently mentioned.

*Health benefits of the exercise.* Across all focus groups, common health benefits gained from EF were improvement in diseases (e.g., hypertension, diabetes, dyslipidemia, and arthritis), muscle strength, flexibility, balance, and well-being. Most participants discussed how their health had been improved after participating in the exercise classes. Other benefits were losing weight; not getting sick as often; improving cholesterol, blood pressure, diabetes, and pain; improving endurance; and sleeping better. One participant with arthritis said, "Exercise helps me feel less limited."

*Mental stimulation.* A theme mentioned by all groups was the cognitive benefits gained from the EF classes: clear thinking, improved memory, and mental well-being. A Chinese participant said, "I come here for happiness, more interesting when there are so many people, more meaningful." Another offered, "I think [exercise] helps us both physically and mentally and spiritually in every way, because to be able to socialize with people and to communicate and have the laughter helps the body to be better and your mind to be stronger." Another participant noted that the exercise class could help prevent depression: "It's really fun and the people you're with crack you up all the time, so no depression here."

## Discussion

This qualitative study of six focus groups was conducted to explore the exercise experience of ethnic older adults in EF, a group-based community exercise program. An ecologic model (13) was used to explore physical environment, social and cultural environment, and individual factors that influenced adherence to exercise. Design of the program is a new factor that emerged from the data. Four main findings resulted from this study, and strategies for promoting exercise adherence among ethnic older adults were generated from the findings. We integrated the factors that participants perceived as beneficial into practical strategies for community leaders or program developers. These strategies were proposed especially for promoting exercise adherence in older adults from ethnically diverse communities and have been provided for EF program evaluation and development (Table 5).

### Sharing culture

Participants in the two Chinese focus groups talked about the importance of sharing the Cantonese language and specifically attended this class because it was all Chinese. This finding is similar to that of Belza et al (17), in which ethnic older adults from focus groups recommended culture-specific exercise programs, sharing culture and language, recruiting an instructor who speaks the language of the group, and weaving components of the culture into the program. The church location for the Chinese EF class was not only a positive physical environment factor but also motivated Chinese participants to attend because church was part of their life. This finding is supported by previous studies that churches are good settings to start new exercise programs for members (27-29).

### Social support

Participants in all focus groups gained social support from exercising in a class with their peers and for this reason looked forward to continuing. There was a sense of strong group cohesion, "a dynamic process reflected by the tendency of a group to stick together and remain united in the pursuit of its instrumental objective and/or for the satisfaction of member affective needs" (29, p. 230). Participants' commitment to their classes came from enjoyment of the class, the instructor, and each other.

Families were important to exercise adherence. Many adult children reportedly helped the participants get to class and encouraged them to keep going. This finding is consistent with that of Belza et al (17), who showed that family encouragement to be physically active is important.

### Physical and mental health outcomes

Positive physical and mental health outcomes gained from the EF classes were powerful motivators to adherence. Participants identified multiple health benefits that they had gained from attending EF, including improvements in strength, balance, endurance, flexibility, and chronic diseases. They also attributed cognitive benefits and mental well-being to participation in EF. Published studies do not indicate the importance of findings such as these to ethnic adult participants.

### Personality traits, characteristics, and feelings

Our findings suggest that many personality traits, characteristics, or feelings are important to ethnic older adults' adherence to exercise. Being competitive, liking structure in daily life, wanting to maintain independence, enjoying and seeking happiness, being sociable, and being accountable were identified as helpful to adherence, as was having perseverance, a commitment to exercise, a guilty conscience, a sense of humor, a positive attitude, pride, and confidence. Self-efficacy is one of the most studied personality traits contributing to adherence to exercise (9,14,18).

A major strength of this study was that data were obtained directly from ethnic older adults and that they had an opportunity to describe their experiences in their own words. Notably, however, the participants were self-selected and thus might represent a more highly motivated group of older adults. Findings from this study, therefore, have limited application to ethnic older adults who drop out of exercise classes and to those who are less social but still like to exercise. Another limitation was the three different focus group facilitators; data obtained during the focus groups might have varied because of different facilitation styles. Lastly, one to two focus groups for each ethnic group is a small sample; additional focus groups should be conducted using a similar interview guide with older adults from the same ethnic communities.

Future research might address the following questions: What is the relationship of group cohesion to adherence? What is the role of family in exercise maintenance by ethnic older adults? How do personality traits and characteristics, other than self-efficacy, influence adherence? What kind of exercise program design increases adherence? How do health care providers' recommendations or referrals affect the commitment of a patient or client to a fitness program? This study supports the roles of the physical environment, design of the program, social environment, and individual biology and behavior in adherence to an exercise program, and suggests strategies for community-based physical activity programs for older adults from ethnically diverse communities. 

## Figures and Tables

**Table 1:** Focus Group Questionnaire, EnhanceFitness Program

**Question**	**Probe**
Tell us your name and briefly describe your experience in this program.	Family, friends, spouse, pastor, church, health care provider, benefits of exercise, location, like to exercise, beliefs, spiritual beliefs.
How did you find out about this exercise program?	
Why did you initially attend this program?	
What keeps you coming back to this program?	Physical health (muscle strength, balance), mental health (mood), friends, location, class time, personality, feel good, beliefs, spiritual beliefs.
If you missed any classes, what were the reasons?	Transportation, time conflict, location, not motivated.
What are the benefits to you for participating in this exercise program?	Physical health (muscle strength, balance), mental health (mood), friends.
Have you participated in other types of physical activity? If yes, what were they? What are the differences between exercise in this program and your other experiences (past experiences)?	Transportation, location, class time.
What environmental factors encourage you to attend or not to attend this program?	
How do environmental factors affect your attitude to attend this program?	
What personality characteristics do you have that help you to exercise?	Like to plan ahead, prefer to be spontaneous, confidence (self-efficacy).
What changes to this program would help you to continue to participate in it?	Location of the class, class content.

**Table 2:** Demographics and Characteristics of Participants^a^ in Study of Ethnic Older Adults (N = 52), EnhanceFitness Program, Seattle, Washington, 2005

Demographic or Characteristic	N (%)
**Sex**	
Female	44 (85)
Male	8 (15)
**Race or ethnicity**	
Chinese	21 (40)
African American	18 (35)
White	10 (19)
Japanese	3 (6)
**Religion**	
Christian	36 (69)
Buddhist	5 (10)
Catholic	4 (8)
Jewish	4 (8)
Other	2 (4)
Atheist	1 (2)
**Frequency of EF participation**	
About 3 times a week	30 (58)
About 2 times a week	15 (29)
About once a week	5 (10)
Less than once a week	0
Missing data	2 (4)
**How confident are you that you will continue to attend this exercise program over the next 6 months?**	
Completely	39 (75)
Almost totally	8 (15)
Quite a bit	2 (4)
Moderately	1 (2)
Slightly	0
Not at all (0)	0
Missing	2 (4)

EF indicates EnhanceFitness Exercise Program.

a Mean (SD) age = 76.8 years (7.4); range, 62-96 years. Mean (SD) duration of EF participation = 44 months (37.8); range, 2-96 months.

**Table 3:** Demographics and Characteristics of Participants by Focus Group (N = 52) in Study of Ethnic Older Adults, EnhanceFitness Program, Seattle, Washington, 2005

Demographic or Characteristic	Focus Group					

	Chinese Baptist Church		Senior Centers With Mixed Ethnicities			

	n = 8	n = 10	n = 8	n = 8	n = 9	n = 9
**Ethnicity**						
Chinese	8	10	1		2	
African American			2	8		8
White			3		6	1
Japanese			2		1	
**Sex**						
Female	7	8	7	7	6	9
Male	1	2	1	1	3	0
**Mean (SD) duration of EF participation, in months**	7 (1)	7 (2)	62 (19)	65 (27)	53 (46)	79 (26)
**Confidence for participation over next 6 months^a^**	4	5	5	5	5	5

EF indicates EnhanceFitness Exercise Program.

a 0 = not at all, 5 = completely.

**Table 4:** Categories and Themes That Influence Exercise Adherence in Study of Ethnic Older Adults, EnhanceFitness Program, Seattle, Washington, 2005

Category	Definition	Themes and Subthemes
**Physical environment**	Settings in which people live, exercise, and interact	Convenient location: senior centers and churches Transportation, weather, and facility
**Design of the EF Program**	Characteristics of EF program such as exercise content and program delivery	Design of the exercise content: variety of exercise, complete body workout Program delivery: morning classes, free or low cost, using weights, and physical performance evaluation
**Social environment**	Interactions with family, friends, health care providers, instructors, and other social networks. Includes cultural customs such as language and religion	Socializing: being with peers, main social outlet, sharing rides and calls Support and influence from family: rides and encouragement Health care provider support: encouragement, referrals, or both Instructor's encouragement, personality, and training Culture-specific factor: shared language and religion
**Individual biology and behavior**	Each person's traits, characteristics, feelings, past experience, and biology (genetics, physical and mental health)	Personality traits and characteristics: being competitive, perseverance, positive attitude, commitment, sense of humor, independence, confidence, seeking to be happy, liking to have structure in daily life Personal feelings: boredom, guilty conscience, pride, and wanting to feel happy Past physical activity experiences Health benefits: improved chronic diseases (dyslipidemia, diabetes, hypertension, and arthritis), improved flexibility, strength, balance, and well-being Mental stimulation: clear thinking, improved memory, and mental well-being

EF indicates EnhanceFitness Exercise Program.

**Table 5:** Strategies to Promote Exercise Adherence in Older Adults From Ethnically Diverse Communities

Categories	Strategies
**Physical environment**	Use existing community settings such as churches and senior centers to offer programs. Have the class location on the main bus lines.
**Design of the EF program**	Design the exercise program content to fit older adults' health needs. Make the exercise content easy for older adults to follow. Consider having the classes in the morning. Use the physical evaluation of progress to show older adults their improvement. Encourage them to set new goals.
**Social environment**	Encourage older adults to join a group exercise program for socializing and social support. Have families frequently check with older adults and encourage them to exercise. Have health care providers give a list of local exercise resources to their clients. Ask health care providers to encourage older adults to exercise by emphasizing the health benefits, monitoring progress, and giving feedback on health improvements to patients. Offer culture-specific classes taught by an instructor who shares the language of the group. Carefully choose and train instructors according to older adults' needs because they are the main reason that older adults stay in the program.
**Individual biology and behavior**	Encourage older adults to join an exercise program to maintain an independent lifestyle. Emphasize the benefits of joining an exercise program as having a routine of life. Ask exercise class to share with other people the health benefits and enjoyment they received. Encourage older adults to use their unique personality traits to help them to exercise.

## References

[1] Gillespie LD, Gillespie WJ, Robertson MC, Lamb SE, Cumming RG, Rowe BH (2003). Interventions for preventing falls in elderly people. Cochrane Database Syst Rev.

[2] Taylor AH, Cable NT, Faulkner G, Hillsdon M, Narici M, Van Der Bij AK (2004). Physical activity and older adults: a review of health benefits and the effectiveness of interventions. J Sports Sci.

[3] Cassidy K, Kotynia-English R, Acres J, Flicker L, Lautenschlager NT, Almeida OP (2004). Association between lifestyle factors and mental health measures among community-dwelling older women. Aust N Z J Psychiatry.

[4] Laurin D, Verreault R, Lindsay J, MacPherson K, Rockwood K (2001). Physical activity and risk of cognitive impairment and dementia in elderly persons. Arch Neurol.

[5] Schuit AJ, Feskens EJ, Launer LJ, Kromhout D (2001). Physical activity and cognitive decline: the role of the apolipoprotein e4 allele. Med Sci Sports Exerc.

[6] Deforche B, De Bourdeaudhuij I (2000). Differences in psychosocial determinants of physical activity in older adults participating in organised versus non-organised activities. J Sports Med Phys Fitness.

[7] McAuley E, Blissmer B, Marquez DX, Jerome GJ, Kramer AF, Katula J (2000). Social relations, physical activity, and well-being in older adults. Prev Med.

[8] Brawley LR, Rejeski WJ, King AC (2003). Promoting physical activity for older adults: the challenges for changing behavior. Am J Prev Med.

[9] King AC (2001). Interventions to promote physical activity by older adults. J Gerontol A Biol Sci Med Sci.

[10] Kriska AM, Rexroad AR (1998). The role of physical activity in minority populations. Womens Health Issues.

[11] Conn VS, Minor MA, Burks KJ, Rantz MJ, Pomeroy SH (2003). Integrative review of physical activity intervention research with aging adults. J Am Geriatr Soc.

[12] Conn VS, Tripp-Reimer T, Maas MS (2003). Older women and exercise: theory of planned behavior beliefs. Public Health Nurs.

[13] (2000). Healthy People 2010: understanding and improving health.

[14] Satariano WA, McAuley E (2003). Promoting physical activity among older adults: from ecology to the individual. Am J Prev Med.

[15] Sallis JF Bauman A Pratt M 15 4 1998 379 397 Environmental and policy interventions to promote physical activity Am J Prev Med Sallis JF, Bauman A, Pratt M. Environmental and policy interventions to promote physical activity. Am J Prev Med 1998;15(4):379-97. 9838979 10.1016/s0749-3797(98)00076-2

[16] Wallace J Buchner D Grothaus L Leveille S Tyll L LaCroix A 53A 4 1989 M301 M306 Implementation and effectiveness of a community-based health promotion program for older adults J Gerontol Wallace J, Buchner D, Grothaus L, Leveille S, Tyll L, LaCroix A. Implementation and effectiveness of a community-based health promotion program for older adults. J Gerontol 1989;53A(4):M301-6. 10.1093/gerona/53a.4.m301 18314570

[17] Belza B, Walwick J, Shiu-Thornton S, Schwartz S, Taylor M, LoGerfo J (2004). Older adult perspectives on physical activity and exercise: voices from multiple cultures. Prev Chronic Dis.

[18] McAuley E, Jerome GJ, Marquez DX, Elavsky S, Blissmer B (2003). Exercise self-efficacy in older adults: social, affective, and behavioral influences. Ann Behav Med.

[19] Schoster B, Callahan LF, Meier A, Mielenz T, DiMartino L (2005). The People with Arthritis Can Exercise (PACE) program: a qualitative evaluation of participant satisfaction. Prev Chronic Dis.

[20] Bandura A (1977). Self-efficacy: toward a unifying theory of behavioral change. Psychol Rev.

[21] Schutzer KA, Graves BS (2004). Barriers and motivations to exercise in older adults. Prev Med.

[22] Greene BL, Haldeman GF, Kaminski A, Neal K, Lim SS, Conn DL (2006). Factors affecting physical activity behavior in urban adults with arthritis who are predominantly African-American and female. Phys Ther.

[23] Melillo KD, Williamson E, Houde SC, Futrell M, Read CY, Campasano M (2001). Perceptions of older Latino adults regarding physical fitness, physical activity, and exercise. J Gerontol Nurs.

[24] King AC, Rejeski WJ, Buchner DM (1998). Physical activity interventions targeting older adults. A critical review and recommendations. Am J Prev Med.

[25] Belza B, Shumway-Cook A, Phelan E, Williams B, LoGerfo J (2006). The effects of a community-based exercise program on function and health in older adults: the EnhanceFitness Program. J Appl Gerontol.

[26] Polit DF, Beck CT (2004). Nursing research: principles and methods.

[27] Wilcox S, Oberrecht L, Bopp M, Kammermann SK, McElmurray CT (2005). A qualitative study of exercise in older African American and white women in rural South Carolina: perceptions, barriers, and motivations. J Women Aging.

[28] Izquierdo-Porrera AM, Powell CC, Reiner J, Fontaine KR (2002). Correlates of exercise adherence in an African American church community. Cultur Divers Ethnic Minor Psychol.

[29] Estabrooks PA, Carron AV (2000). The Physical Activity Group Environment Questionnaire: an instrument for the assessment of cohesion in exercise classes. Group Dyn.

