# Erratum, Vol. 5: Issue 1

**Published:** 2008-03-15

In the article "'It Is Our Exercise Family': Experiences of Ethnic Older Adults in a Group-Based Exercise Program," the e-mail address of the corresponding author, Basia Belza, was incorrect. The correct e-mail address is basiab@u.washington.edu. Corrections were made on our Web site on December 19, 2007, and appear online at www.cdc.gov/pcd/issues/2008/jan/06_0170.htm. We regret any confusion or inconvenience this error may have caused.

